# Wine aging: a bottleneck story

**DOI:** 10.1038/s41538-019-0045-9

**Published:** 2019-08-06

**Authors:** Thomas Karbowiak, Kevin Crouvisier-Urion, Aurélie Lagorce, Jordi Ballester, André Geoffroy, Chloé Roullier-Gall, Julie Chanut, Régis D. Gougeon, Philippe Schmitt-Kopplin, Jean-Pierre Bellat

**Affiliations:** 1 Univ. Bourgogne Franche-Comté, AgroSup Dijon, PAM UMR 02 102, 1 Esplanade Erasme, 21000 Dijon, France; 2 Univ. Bourgogne Franche-Comté, Laboratoire Interdisciplinaire Carnot de Bourgogne, UMR 6303 CNRS, 9 Avenue Alain Savary, 21000 Dijon, France; 3 Univ. Bourgogne Franche-Comté, Institut Universitaire de la Vigne et du Vin, 1 rue Claude Ladrey, 21000 Dijon, France; 4 13 rue du 8 mai 1945, 21220 Brochon, France; 5 Research Unit Analytical BioGeoChemistry, Department of Environmental Sciences, Helmholtz Zentrum München, Ingolstaedter Landstr. 1, 85764 Neuherberg, Germany; 6 TUM Technische Universität München, Analytical Food Chemistry, Platform Maximus-von-Imhof-Forum 2, 85354 Freising, Germany

**Keywords:** Materials science, Engineering, Agriculture

## Abstract

The sporadic oxidation of white wines remains an open question, making wine shelf life a subjective debate. Through a multidisciplinary synoptic approach performed as a remarkable case study on aged bottles of white wine, this work unraveled a yet unexplored route for uncontrolled oxidation. By combining sensory evaluation, chemical and metabolomics analyses of the wine, and investigating oxygen transfer through the bottleneck/stopper, this work elucidates the importance of the glass/cork interface. It shows unambiguously that the transfer of oxygen at the interface between the cork stopper and the glass bottleneck must be considered a potentially significant contributor to oxidation state during the bottle aging, leading to a notable modification of a wine’s chemical signature.

## Introduction

Because of its universally shared cultural heritage, wine constitutes an emblematic consumer experience, driving curiosity, and excitement far beyond that of other food products. This is typified by the process of cellar aging, during which great wines are supposed to experience consecutive developmental stages to reach a climax after years or even decades. While most associated with red wines, the mystique of aging also applies to white wines. Their aging mechanisms related to oxidation have been of particular interest. During cellar aging, oxidation relates to chemical autoxidation promoted by oxygen ingress into the wine. The subsequent mechanisms involving the chemistry of antioxidants—whether they are intrinsic or added to the wine—have been the subject of various studies since the late 1970s.^1–5^

Since the beginning of the 90s, the worldwide problem of premature oxidation of white wines has further stressed the need for better understanding of the complex chemical interplay involved in oxygen consumption,^6–8^ and the need for advanced tools to predict the aging ability of white wines.^9–11^ With respect to wine bottle aging during wine storage, the stopper constitutes the last rampart that preserves wine from oxygen ingress. This is the reason why many studies have focused on the gas barrier properties of the different types of stoppers since the mid-90s, comparing natural corks of different quality, agglomerated corks, synthetic stoppers, and screwcaps.^12–17^ Very recently, many different analyses methods were critically assessed and reviewed, along with permeability and diffusion data.^14^ From all the scientific articles dedicated to the permeability of wine stoppers and wine oxidation, it is noteworthy that only a very few investigated the correlation between sensory evolution of wine and the permeability of the stopper.^18–21^

Although there are obvious differences in oxygen permeability among the main closure types, these differences do not explain why uncontrolled oxidation can sporadically occur, and leaves the role the stopper may play in question. Compared to other bottled products, this is particularly relevant for wine, where shelf life can be extremely long and very difficult to determine.

Used to seal amphorae since the age of the Romans, cork still accounts for approximately two thirds of the wine stopper market. Surprisingly, although it is used as a sealant, the diffusion coefficient of oxygen in cork has only been determined recently.^22^ As a natural material, cork is also well-known as one of the first systems depicted under the microscope, and origin of the name “cell” was first given to the basic biological unit observed in cork.^23^ Cork is an alveolar material,^24^ composed of empty cells several tens of microns wide, arranged in a honeycomb pattern, and separated by a cell wall of about a micron thick.^25^ It also exhibits macroporosity due to lenticular channels, which is used as the industrial measure to classify cork quality.

The limiting step of gas transfer in cork is the diffusion across the cell wall.^26^ The mean value of the effective diffusion coefficient in a full cork stopper (non-compressed) is around 10^−9^ m^2^ s^−1^, with a statistical distribution ranging from 10^−10^ to 10^−8^ m^2^ s^−1^.^22^ When compressing cork, in the range of compression used for still wines (a reduction of 23% in diameter and 40% in volume), the diffusion coefficient remains in the same order of magnitude as noncompressed cork.^27^ However, when inserted in the neck of a bottle, the transfer occurring at the interface between the glass and the stopper may significantly contribute to oxygen ingress into the bottle. This was noticed in the particular case of a gradient-imposed diffusion of oxygen for a dry cork without surface treatment inserted in a glass bottleneck, using 200 hPa and ∼0 hPa of oxygen pressure on both sides of the cork sample, leading to a 50× increase in the effective diffusion coefficient.^27^ The effect of environmental parameters, such as the presence of water and ethanol, as well as the role of cork surface coatings, are further factors to assess in order to better understand the interface transfer. Nevertheless, the interface between the glass bottleneck and the stopper could represent a preferential pathway for gases.

In the present work, the question of oxidative stability during bottle aging and the related question of oxidative stability of dry white wines have been considered. The objective was to characterize the contribution of stoppers on bottle aging of white wines in real condition, with particular emphasis on the bottleneck/stopper interface. Sporadic oxidation was observed in a few, but not all, bottles of white wine coming from the same vintage and production lot, i.e., visual examination showed obvious color evolution. To investigate this phenomenon, a multidisciplinary synoptic approach was designed combining sensory evaluation, targeted and non-targeted chemical analyses, and physical investigation of both the wine and the system composed of the stopper and the bottleneck.

## Results and discussion

### Sensory evaluation of the wines

**Fig. 1:**
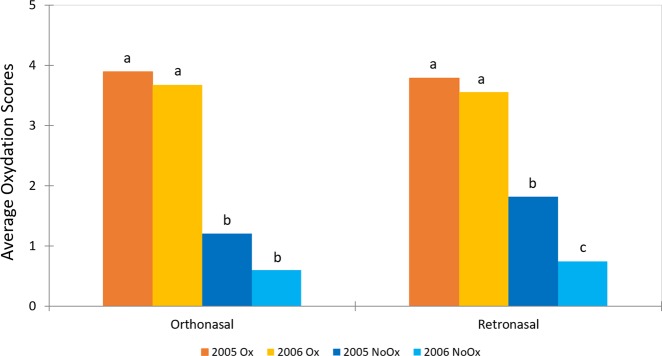
Average oxidation score of the four wines, evaluated orthonasally (left) and retronasally (right). Means with the same letter are not significantly different according to Newman–Keuls test (*⍺* = 0.05)

The analysis of variance (ANOVA) carried out on the sensory data showed a significant sample effect for both orthonasal (*F* = 4.27; *p* < 0.0001) and retronasal (*F* = 5.35; *p* < 0.0001) oxidative notes. The average oxidation scores and the results of the Newman–Keuls post-hoc test are reported in Fig. 1. Consistent with enological parameters (see below), the sensory results clearly showed that, for both vintages (2005 and 2006), the wines we suspected being oxidized (Ox) had significantly higher oxidative odors than the ones we suspected were not oxidized (NoOx).

### Enological parameters

The enological parameters of the four wines before bottling and after uncorking are given in Table 1. Despite the fact that analyses of wines before bottling and after uncorking were performed in different laboratories, most parameters showed close agreement, with the notable exception of SO_2_. SO_2_ is known to decrease significantly during the first years, or even months, after bottling because of the consumption of oxygen brought about by the bottling process and/or diffusing through the stopper.^14,28^ Moderate oxygen ingress thus leads to multiple chemical reactions involving SO_2_, including in particular its nucleophilic addition to quinones,^5,6,29^ in which case free SO_2_ is preferentially consumed. In contrast, high oxygen ingress can significantly mobilize both the free and reversibly bound SO_2_.

**Table 1:** Enological parameters of wines, before bottling and after uncorking (in 2016)

Wine enological parameters	2005			2006		
	Before bottling	After uncorking		Before bottling	After uncorking	
		NoOx	Ox		NoOx	Ox
Ethanol concentration (% V/V)	12.90	12.90	13.04	13.05	13.09	13.02
pH	3.35	3.28	3.25	3.26	3.36	3.32
Total acidity (g L^−1^, eq. H_2_SO_4_)	3.10	3.66	3.67	3.37	3.57	3.55
Volatile acidity (g L^−1^)	0.25	0.43	0.43	0.40	0.42	0.41
Total SO_2_ concentration (mg L^−1^)	88	35.8	10.2	71	81.9	10.2
Free SO_2_ concentration (mg L^−1^)	35	7.7	5.1	20	12.8	5.1

The observed decrease of total SO_2_ concentration (and thus of the bound fraction) in the Ox wines, whatever the vintage, is a clear illustration of their likely higher oxygenation undergone during bottle aging (Table 1), consistently with sensory results above.

**Fig. 2:**
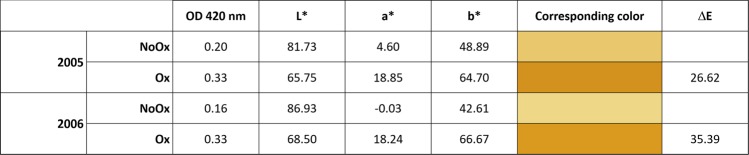
Wine color as measured by OD at 420 nm and L*a*b* (with calculated value of Δ*E*)

An additional consequence of a high oxygenation of white wines is the oxidation of polyphenolic compounds such as epicatechin, which ultimately forms colored pigments.^30^ In such context, the color of wines changes from greenish or pale yellow to brownish, as witnessed in particular by the absorbance at 420 nm. Whether it is the 420 nm increase, the L* coordinate decrease or the a* coordinate increase between NoOx and Ox wines (Fig. 2), our color measurements clearly confirmed that Ox wines were significantly more oxidized than NoOx wines. It must be noted that for the two vintages, Δ*E* values between Ox and NoOx wines largely exceeded the threshold of 3, from which a color difference unambiguously becomes detectable by the human eye.^31^ This first observation was actually the starting point of the selection of these samples within the production batch of the winemaker, this difference being even visible through the bottle glass (Fig. 5).

### Metabolomics analyses

**Fig. 3:**
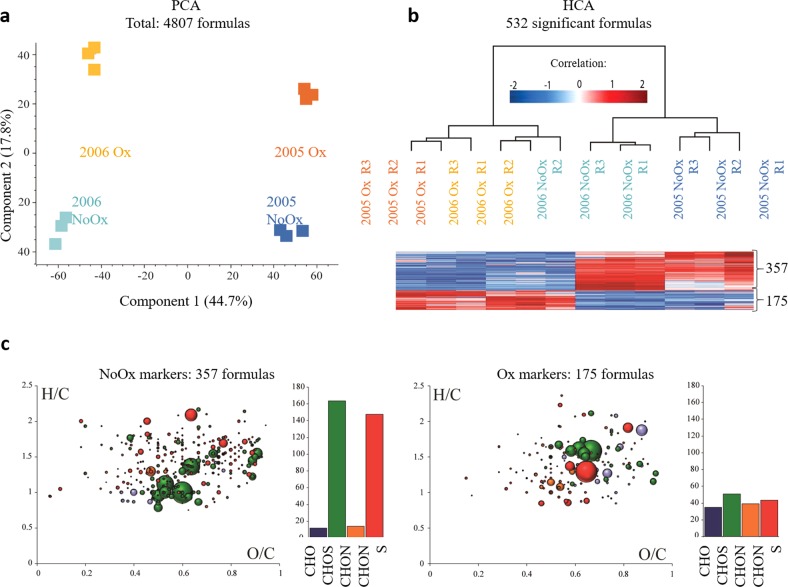
Metabolomics analyses of wine. **a** Principal component analysis of FT-ICR-MS data from the 12 samples (four wines in triplicate). The first two components retained 62.5% of the variation. **b** Hierarchical cluster analysis (HCA) colored according to relative signal intensity, from blue (low) to red (high), verified by ANOVA test and *p*-values < 0.01 (for all four wines and each replicate R1 R2 R3). **c** van Krevelen diagrams (H/C versus O/C) of the elemental formulas showing significantly higher intensity (*p*-values < 0.01) for NoOx wines (left) and Ox wines (right), color code of van Krevelen diagrams: CHO, blue; CHOS, green; CHON, orange; CHONS, red. Bubble sizes indicate relative intensities of corresponding peaks in the spectra

Each of the four wine samples analyzed by FT-ICR-MS, led to the recording of nearly 5000 mass peaks to which an unambiguous elemental formula could be assigned. This total number of elemental formulas was slightly lower (2–3%) for Ox wines. The unsupervised (principal component analysis) PCA statistical analysis unambiguously separated vintages along the first axis, accounting for 44.7% of the variation, whereas oxidation levels clearly separated along the second axis, retaining 17.8% of the variation (Fig. 3a). Further, a standardized dataset (532 masses out of 4807) containing all m/z values detected by FT-ICR-MS which were significantly more intense in Ox or NoOx wines (verified by ANOVA test and *p*-values ≤ 0.01) was used for hierarchical cluster analysis (HCA) of the samples (Fig. 3b) and for van Krevelen diagrams representations (Fig. 3c). Of these 532 masses, 175 m/z values were discriminant for Ox wines, whereas up to 357 m/z values were discriminant for NoOx wines (Fig. 3c). These van Krevelen diagrams, which sort annotated elemental formulas from identified discriminant mass peaks, further revealed that markers for NoOx wines are found in the areas of sulfonated polyphenols, amino acids/peptides, and glycosylated compounds.^32^ A striking feature of NoOx markers is the relatively high contribution of S- and N-containing compounds as revealed by the corresponding CHOS/CHO and CHONS/CHO ratios (16.0 and 14.5, respectively). In contrast, the corresponding ratios for Ox markers (2.0 and 1.6 for CHOS/CHO and CHONS/CHO ratios, respectively) showed a significantly reduced contribution of S- and N-containing compounds, which appear mostly located in the area of amino acids/peptides, but not in the area of polyphenols. Such observations are consistent with the above-mentioned enological analyses, and in particular with SO_2_ concentrations for these wines, which showed that NoOx wines had higher total (and bound) SO_2_ concentrations than Ox wines. As shown recently,^6^ higher levels of bound SO_2_ could be associated with higher relative abundances of CHOS and CHONS compounds. Since such compounds can potentially be involved in the resistance of wines against oxidation,^33^ their relative absence in Ox wines further demonstrates that the latter had encountered high oxygenation and subsequent oxidation levels during bottle aging. It must be noted that the low abundance of the CHOS and CHONS mass peaks in FT-ICR-MS spectra of Ox wines (and in particular those corresponding to sulfonated polyphenols) can be explained by the fact that, upon oxygenation, the latter may react to form more condensed structures, including colored pigments corresponding to the color measurements presented above. Such condensed structures can then disappear from FT-ICR-MS spectra, either because of precipitation or because of a lower ionization efficiency.^34^

### Oxygen permeation in the bottles

**Fig. 4:**
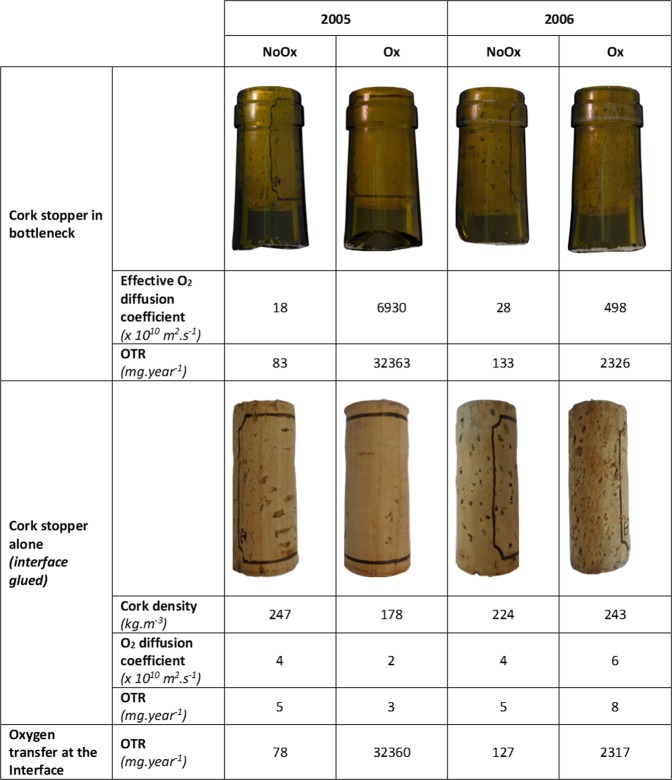
Effective oxygen diffusion coefficient and oxygen transmission rate (OTR) determined for cork stopper in bottleneck and cork stopper alone

Oxygen permeation into bottles was approached in two ways. First, gas transfer was measured through the whole system composed of the cork inserted in the neck of the bottle. The values of the effective diffusion coefficient were clearly higher for the Ox wines than the NoOx ones; as much as 400× higher in the case of the 2005 vintage (Fig. 4). The oxidation of these wines was thus likely due to an uncontrolled transfer of oxygen in the bottle. Second, the gas transfer was measured through the cork stopper itself, once extracted from the bottleneck and inserted, non-compressed, in a metal ring with a glued interface. Under these conditions, the oxygen diffusion coefficient through the cork was approximately the same for the four cork stoppers, with a value similar to those measured on natural cork in previous works.^22,26^ The results point out the role played by the interface between the cork stopper and the glass bottleneck. From these diffusion coefficients, the respective oxygen transmission rates through the cork/bottleneck system, through the cork alone, and by difference through the interface can be calculated. The values, expressed as mg of oxygen per year and per bottle, are given in Fig. 4.

It must be noted that these values were calculated from measurements performed in different conditions than those found in typical wine storage (i.e., a dry sample under vacuum and without partial pressure of water vapor and ethanol). It is also worth noting that these values correspond to the barrier property of cork in its final state after many years of storage, and gas barrier properties may have changed over time. Nevertheless, the data obtained clearly show that the oxygen transfer at the interface between the cork and the bottleneck was always higher than the transfer through the cork alone.

In the case of Ox wines, this transfer at the interface is much greater, up to 10,000× that of the transfer through the cork. For NoOx wines, the transfer at the interface is only 10× higher than the transfer through the cork. Even if there is a high variability of oxygen transfer rate through natural cork (ranging from 0.03 to 271 mg year^−1^),^14^ this cannot account for the high difference observed between Ox and NoOx wines in the present work. The oxidation of wine is thus not due to the low barrier property of the cork, but to an uncontrolled transfer of oxygen at the interface. As previously assumed in a study performed on a statistically representative set of natural cork samples inserted or not in a bottleneck,^27^ this remarkable case study performed on aged bottles of white wine clearly highlights the interface as the preferential pathway for oxygen ingress in the bottle.

Moreover, the diameter of each stopper was measured immediately after cork extraction from the bottleneck and after one month. In all cases, the stoppers did not recover their initial diameter of 24 mm before bottling. The final diameter lay between 19 and 19.5 mm, which corresponds to a recovery of ~80% of the initial value. No change in dimension was noticeable one month after extraction. Considering this, the final cork dimensions led to an overestimation of density; the apparent density is reported in Fig. 4. A surprising result came from the 2005 Ox sample, which was the most Ox wine with the highest gas transfer at the interface, but in which cork stopper presented the best apparent quality. It displayed the lowest surface density of lenticels and the lowest cork density (as shown in Fig. 4). This raises an important question about the real impact of lenticel content, which defines cork quality, has on the gas barrier properties of the cork stopper in the bottleneck.

Wine oxidative stability during bottle aging was thus investigated in this study through a top-down approach combining sensory evaluation, targeted and non-targeted chemical analyses of the wine, and oxygen transfer investigation of the system composed of the stopper and the bottleneck. A few bottles were sampled from the same batch of dry white wine after several years of storage in bottle, using two different vintages, with a standard and a straightforward color evolution presupposing higher oxidation in both cases.

First, both the sensory evaluation and the chemical analyses of classical enological parameters unambiguously revealed the different oxidative states of the four bottles, with, for each vintage, one bottle being oxidized compared to the other. Oxidative notes of Ox wines were clearly identified by a trained panel in orthonasal and retronasal perception. This was also confirmed by the color difference between NoOx and Ox wines, with ∆*E* values above 3, and by the low concentration of total and bound SO_2_ in Ox wines.

Further, a metabolomics analysis was performed by FT-ICR-MS. A total of 532 masses were significantly more intense in Ox or NoOx wines, of which 175 m/z values were distinct for Ox wines and 357 m/z values for NoOx wines. These non-oxidative markers were mainly sulfonated polyphenols, amino acids/peptides, and glycosylated compounds, with a high contribution of S- and N-containing compounds as revealed by the corresponding CHOS/CHO and CHONS/CHO ratios. In contrast, oxidative markers are characterized by a significantly reduced contribution of compounds containing S and N atoms, probably due to a higher oxygenation level.

Lastly, the oxygen transfer rate was first determined through the whole system composed of the glass bottleneck containing the cork stopper, then on the cork stopper alone with the interface glued (after uncorking). The diffusion coefficient of oxygen through the cork stopper alone was similar for all stoppers. However, the transfer of oxygen through the cork/glass bottleneck system was higher than through the cork alone, and much higher for bottles containing the Ox wines. Therefore, this study sheds light on the potential role of the glass/cork interface in the oxidative evolution of the wine during bottle aging. Within the frame of our experimental setup, our results revealed for the first time that the oxidative stability of white wine during bottle aging could be modulated by a pronounced ingress of oxygen at the interface between the cork stopper and the glass bottleneck, independently of the cork stopper quality (which all four displayed low barrier property in this case study). However, considering that several other factors can contribute to the oxidative state and evolution of a wine (including vineyard environmental conditions related to climate change, winemaking practices such as SO_2_ reduction, overall matrix composition, etc.), such multidisciplinary investigation should definitely be extended to more samples, in order to be able to hierarchize contributing factors.

## Methods

### Materials

**Fig. 5:**
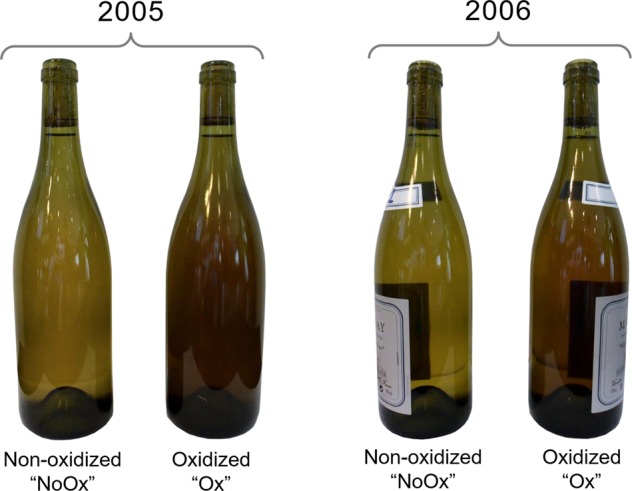
Bottles analyzed in this study, from 2005 to 2006 vintages, originated from the same batch, with one suspected to be non-oxidized and one suspected to be oxidized

Four bottles of white wine from Burgundy (Chardonnay variety, appellation Marsannay) were used for this study (Fig. 5). Two bottles were from the 2005 vintage and the two others from the 2006 vintage. Each vintage bottle was issued from the same 5 hL tank. In each case, one bottle was suspected to be NoOx and the other suspected to be oxidized, due to the color difference visible through the bottle glass.

All stoppers were natural cork of high quality, 24 mm diameter, and 49 mm length. Once all experiments were performed, the bottleneck profiles were measured using a profilometer (Egitron PerfiLab PRF 2014 01). All four bottles were within the tolerances indicated in the standard.^35^

### Sampling protocol

In order to not damage the neck of the bottle containing the stopper, the wine contained in the bottles was sampled, in June 2016, as follows. First, the glass on the side of the bottles was predrilled in two places. Then, in an inert hood under argon, the holes were made, and the wine was filtered to remove any glass debris that may have been there. Part of the wine was used for the sensory analysis described below and the remaining part was used for the chemical analyzes detailed in sections “enological parameters” and “metabolomics analyses”. All the samples taken were blanketed with argon until analysis.

### Sensory evaluation

#### Panel

Fifteen enology students of the “Institut Universitaire de la Vigne et du Vin—Jules Guyot” at Burgundy University were selected among 28 candidates. They were specifically trained on wine oxidation and reduction, following a previously published strategy.^9^ The training process lasted 10 weeks and was carried out in conjunction with their regular tasting lessons within the frame of the enology training, where they learned the main olfactory notes of wine. The main goals of the training were first to familiarize the candidates with reduction and oxidation odors (by using naturally Ox or reduced wines or even spiked wines) and second, to familiarize them with the assessment by using the reduction–oxidation scale (see below). Tasters’ selection was based on their performance during the last three sessions which tested their ability to recognize and score the oxidative notes in wine, their consensus with the rest of the panel and their repeatability. At the end of the process, the selected judges had extensive experience in wine tasting, including descriptive analysis related to wine oxidation.

#### Method

Sensory analysis was carried out in a sensory lab equipped with individual tasting booths. All samples were assessed in a single 1h-session. Samples were served at room temperature in opaque glasses, following a Latin square design. Concerning the presentation order, four different 4 × 4 Latin squares were generated using FIZZ software (Biosystèmes, Couternon, France) yielding 16 balanced presentation orders. We used the first fifteen orders for our experiment.

Panelists were asked to assess reductive and oxidative sensory characters by means of a structured interval scale ranging from −5 (labeled “strong reduction”) to +5 (labeled “strong oxidation”). The zero position was labeled “neither reduced nor oxidized”.^8,9,33^ Each sample was assessed first orthonasally and then retronasally. Participants were asked to rinse their palates with mineral water and unsalted crackers between samples. Each sample was assessed once by each participant.

### Enological parameters

Enological parameters at bottling, provided by the producer, were measured in October 2006 and in May 2008, respectively, for the 2005 and the 2006 vintages. Classical enological analyses of the wines were performed in our laboratory in June 2016, according to OIV technical standards. Enological parameters before bottling and after uncorking were analyzed once for each sample. pH, ethanol concentration, total acidity, and volatile acidity were measured using Fourier Transform InfraRed analysis (OenoFoss, Foss, Hillerød, Denmark). Two-hundred microliters of wine were deposited on the reading cell of the OenoFoss apparatus, after calibration using a standard wine. Before analysis, the wine was centrifuged for 5 min at 10,000 rpm in order to avoid gas bubbles in the liquid phase. After centrifugation, samples were sonicated for 5 min (Bransonic 3210, Branson, Wissous, France) to remove any residual bubbles, and filtered (porosity of 8–11 µm). SO_2_ titration was also performed, using colorimetric titration by the Ripper method. Free and total SO_2_ concentrations were determined.

Absorbance at 420 nm, corresponding to yellow intensity, was measured with a Shimadzu UV-1800 spectrophotometer. Further color absorbance measurements were performed with a Konica Minolta CM-5 spectrophotometer using optical glass precision cells of 50 mm path length (Hellma Analytics) and scanned over the range of 740–360 nm (visible range). Black and white calibrations were performed using a standard black plate and an empty glass cell, respectively. The color was recorded using the CIE-L* a* b* uniform color space (CIE-Lab), using three dimensions (L*, a*, b*) of the Hunter color scale, where L* ranges from 0 for black to +100 for white, a* ranges from −50 for green to +50 for red, and b* ranges from −50 for blue to +50 for yellow. Perfomed in triplicate, color measurement gave standard deviation lower than 0.03 for all analyzed parameters.

### Metabolomics analyses

Ultrahigh resolution mass spectra were acquired using an FT-ICR-MS instrument (solariX, Bruker Daltonik, Bremen, Germany) equipped with a 12 Tesla superconducting magnet and an Apollo II electrospray ionization source operated in negative ionization mode.^32,36,37^ Fifty microliters of each wine were diluted in 1 mL of methanol. Diluted samples were introduced into the micro electrospray source at a flow rate of 120 µLh^−1^ using a syringe pump. Each diluted sample was injected three times. The MS was externally calibrated on clusters of arginine (10 ppm) in methanol. Spectra were acquired with a time domain of four mega-words per second with a mass range from m/z 100 to 1000 Da.^32,38,39^ A total of 400 scans per sample were accumulated. Spectra were internally recalibrated with a reference list including fatty acids and recurrent wine compounds up to m/z 1000, with mass errors below 50 ppb.

The m/z peaks with a signal-to-noise ratio (S/N) of 4 and higher were exported to peak lists. All generated formulas were validated by setting plausible chemical constraints, including isotopic pattern search, N rule, O/C ratio ≤ 1, H/C ratio ≤ 2, element counts: C ≤ 100, H ≤ 200, O ≤ 80, N ≤ 3, S ≤ 3, and P ≤ 1.

### Oxygen permeation

Oxygen permeation measurements were performed in two steps: first, on the cork still inserted in the bottleneck, which was cut from the bottle, and second, on the cork alone (both cases shown in Fig. 4). For the latter, corks were inserted non-compressed in a metal tube and secured with an impermeable epoxy glue to avoid mass transfer at the interface.

The method used to measure oxygen transfer is based on manometry using a homemade device. The equipment and the procedure used to determine the effective diffusion coefficient have been described in detail in previous works.^22,26^ In short, it is based on the knowledge of the oxygen sorption isotherm and on an analytical solution to Fick’s law applied to the steady state. For the present work, the initial pressure of oxygen was fixed close to 1000 hPa on one side of the sample, while the other side was maintained under dynamic vacuum (0.1 hPa). Temperature was kept constant at 298 K (±1 K). Prior to experiment, samples were outgassed in situ during 48 h in order to compare the diffusion coefficient of materials having the same hydration state.

### Statistical analysis

Statistical analyses were performed on each set of data. An ANOVA with samples and panelists as main factors was performed on the oxidation sensory ratings. A Newman–Keuls test was performed when the sample factor was significant. Raw FT-ICR-MS data were first aligned in order to discover underlying patterns, to identify outliers, to reduce the dimensionality of the data, and also to compress large datasets into smaller and more discernible ones.^40^ Peak alignment and filtering of masses were performed in MS Excel 2010 (Microsoft, Redmond, USA) with maximum error thresholds of 1 ppm, and filtered for masses occurring in minimum of 10% of all samples.^38,40^ PCA was performed with Simca-P 9.0 (Umetrics, Sweden) and HCA with Perseus 1.5.1.6 (http://www.perseus-framework.org, Max Planck Institute of Biochemistry, Germany).

## Data Availability

All data are included in this article.
